# *N*-[7-Eth­oxy-2-(prop-2-en-1-yl)-2*H*-indazol-6-yl]-4-methyl­benzene­sulfonamide

**DOI:** 10.1107/S1600536811045855

**Published:** 2011-11-05

**Authors:** Najat Abbassi, El Mostapha Rakib, Abdellah Hannioui, Hafid Zouihri

**Affiliations:** a Laboratoire de Chimie Organique et Analytique, Université Sultan Moulay Slimane, Faculté des Sciences et Techniques, Béni-Mellal, BP 523, Morocco; b Laboratoires de Diffraction des Rayons X, Centre Nationale pour la Recherche Scientifique et Technique, Rabat, Morocco

## Abstract

In the title compound, C_19_H_21_N_3_O_3_S, the C—SO_2_—NH—C torsion angle is 66.20 (9)°. The dihedral angle between the benzene ring and the essentially planar indazole ring system [r.m.s. deviation = 0.0361 (1) Å] is 72.97 (6)°. The S atom has a distorted tetra­hedral geometry [maximum deviation = O—S—O = 119.30 (6)°]. The crystal structure features inversion-related dimers linked by pairs of N—H⋯O hydrogen bonds. In addition, weak C—H⋯O inter­actions may stabilize the crystal packing.

## Related literature

For related structures, see: Abbassi *et al.* (2011*a* ▶,*b* ▶). For the biological activity of sulfonamides, see: Soledade *et al.* (2006 ▶); Lee & Lee (2002 ▶). For the synthesis of 7-eth­oxy-*N*-alkyl­indazole derivatives, see: Abbassi *et al.* (2011*c* ▶).
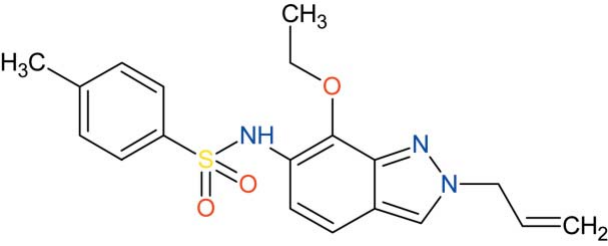


## Experimental

### 

#### Crystal data

C_19_H_21_N_3_O_3_S
*M* *_r_* = 371.45
Monoclinic, 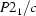

*a* = 10.1459 (7) Å
*b* = 9.9506 (7) Å
*c* = 18.3720 (13) Å
β = 95.097 (3)°
*V* = 1847.5 (2) Å^3^
*Z* = 4
Mo *K*α radiation
μ = 0.20 mm^−1^
*T* = 296 K
0.32 × 0.31 × 0.24 mm

#### Data collection

Bruker APEXII CCD detector diffractometer
20488 measured reflections
4034 independent reflections
3693 reflections with *I* > 2σ(*I*)
*R*_int_ = 0.031

#### Refinement

*R*[*F* ^2^ > 2σ(*F* ^2^)] = 0.036
*wR*(*F* ^2^) = 0.100
*S* = 1.06
4034 reflections
238 parameters
1 restraint
H-atom parameters constrained
Δρ_max_ = 0.69 e Å^−3^
Δρ_min_ = −0.39 e Å^−3^

### 

Data collection: *APEX2* (Bruker, 2005 ▶); cell refinement: *SAINT* (Bruker, 2005 ▶); data reduction: *SAINT*; program(s) used to solve structure: *SHELXS97* (Sheldrick, 2008 ▶); program(s) used to refine structure: *SHELXL97* (Sheldrick, 2008 ▶); molecular graphics: *PLATON* (Spek, 2009 ▶); software used to prepare material for publication: *publCIF* (Westrip, 2010 ▶).

## Supplementary Material

Crystal structure: contains datablock(s) I, global. DOI: 10.1107/S1600536811045855/bt5700sup1.cif

Structure factors: contains datablock(s) I. DOI: 10.1107/S1600536811045855/bt5700Isup2.hkl

Supplementary material file. DOI: 10.1107/S1600536811045855/bt5700Isup3.cml

Additional supplementary materials:  crystallographic information; 3D view; checkCIF report

## Figures and Tables

**Table 1:** Hydrogen-bond geometry (Å, °)

*D*—H⋯*A*	*D*—H	H⋯*A*	*D*⋯*A*	*D*—H⋯*A*
N1—H1*N*⋯O3^i^	0.86	2.21	3.0199 (15)	159
C8—H8⋯O2^ii^	0.93	2.44	3.1270 (17)	131

Symmetry codes: (i) 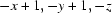; (ii) 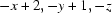.

**Fig. 1.**
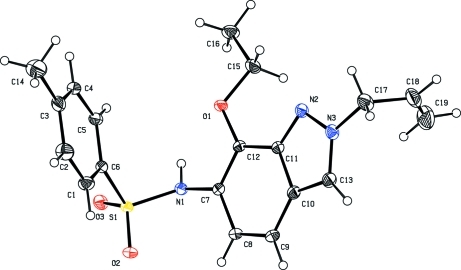
Molecular view of the title compound showing the atom-labeling scheme. Displacement ellipsoids are drawn at the 30% probability level. H atoms are represented as small spheres of arbitrary radii.

**Fig. 2.**
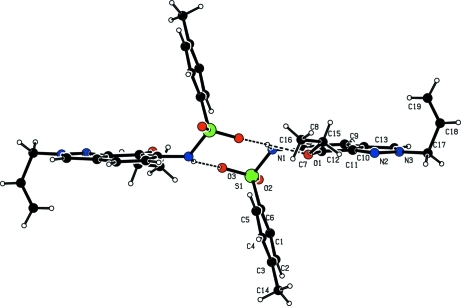
View of the N—H···O bonded dimers of the title compound.

**Fig. 3.**
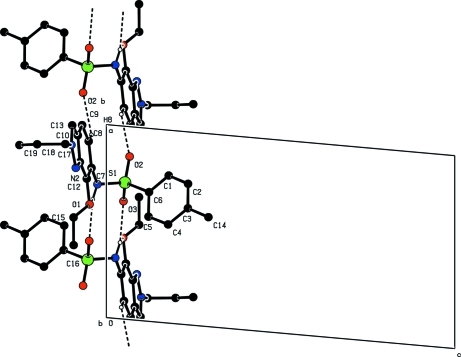
Partial packing view showing N—H···O and C-H···O hydrogen bonds. H atoms not involved in hydrogen bonds have been omitted for clarity.

## supplementary crystallographic information

### Comment

Various sulfonamides are widely used as anti-hypertensive (Soledade *et al.*, 2006; Lee & Lee, 2002). In former papers, we reported the crystal structures of *N*-(7-ethoxy-1*H*-indazol-4-yl)-4-methylbenzenesulfonamide (Abbassi *et al.*, 2011*a*) and *N*-[7-ethoxy-1-(prop-2-en-1-yl)-1*H*-indazol-4-yl]-4-methylbenzenesulfonamide (Abbassi *et al.*, 2011*b*). In this communication, the crystal structure of *N*-[7-ethoxy-2-(prop-2-en-1-yl)-2*H*-indazol-6-yl]-4-methylbenzenesulfonamide is reported.

In the title compound, C_19_H_21_N_3_O_3_S, the C—SO_2_—NH—C torsion angle is 66.20 (9)°. The dihedral angle between the aromatic ring and the indazol system is 72.97 (6)°. The S atom has a distorted tetrahedral geometry [maximum deviation: O—S—O = 119.3  (6)°] (Fig. 1).

Two neighbouring molecules generate a hydrogen-bonded dimer about a center of inversion through a pair of intermolecular N—H···O interactions   (Figs. 2 and 3).

### Experimental

A mixture of 2-allyl-6-nitro-2*H*-indazole [Abbassi *et al.*, 2011*c*] (1.22 mmol) and anhydrous SnCl_2_ (1.1 g, 6.1 mmol) in 25 mL of absolute ethanol was heated at 60 °C for 2 h. After reduction, the starting material disappeared, and the solution was allowed to cool down. The pH was made slightly basic (pH 7–8) by addition of 5% aqueous potassium bicarbonate before extraction with ethyl acetate. The organic phase was washed with brine and dried over magnesium sulfate. The solvent was removed to afford the amine, which was immediately dissolved in pyridine (5 ml) and then reacted with 4-methylbenzenesulfonyl chloride (0.26 g, 1.25 mmol) at room temperature for 24 h. After the reaction mixture was concentrated *in vacuo*, the resulting residue was purified by flash chromatography (eluted with Ethyl acetate: Hexane 1:9).

### Refinement

The H atoms bound to C were positioned geometrically and constrained to ride on their parent atoms [C—H distances are 0.93Å for CH groups with *U*_iso_(H) = 1.2 *U*_eq_(C,N), and 0.97 Å for CH_3_ groups, and the coordinates for the H atom bonded to N were taken from a difference map, and the atom was refined using a riding model.

### Crystal data

**Table:**

C_19_H_21_N_3_O_3_S	*F*(000) = 784
*M**_r_* = 371.45	*D*_x_ = 1.335 Mg m^−^^3^
Monoclinic, *P*2_1_/*c*	Mo *K*α radiation, λ = 0.71073 Å
Hall symbol: -P 2ybc	Cell parameters from 243 reflections
*a* = 10.1459 (7) Å	θ = 2.3–27.2°
*b* = 9.9506 (7) Å	µ = 0.20 mm^−^^1^
*c* = 18.3720 (13) Å	*T* = 296 K
β = 95.097 (3)°	Prism, colourless
*V* = 1847.5 (2) Å^3^	0.32 × 0.31 × 0.24 mm
*Z* = 4	

### Data collection

**Table:**

Bruker APEXII CCD detector diffractometer	3693 reflections with *I* > 2σ(*I*)
Radiation source: fine-focus sealed tube	*R*_int_ = 0.031
graphite	θ_max_ = 27.0°, θ_min_ = 2.0°
ω and φ scans	*h* = −12→12
20488 measured reflections	*k* = −12→12
4034 independent reflections	*l* = −22→23

### Refinement

**Table:**

Refinement on *F*^2^	Secondary atom site location: difference Fourier map
Least-squares matrix: full	Hydrogen site location: inferred from neighbouring sites
*R*[*F*^2^ > 2σ(*F*^2^)] = 0.036	H-atom parameters constrained
*wR*(*F*^2^) = 0.100	*w* = 1/[σ^2^(*F*_o_^2^) + (0.0522*P*)^2^ + 0.8814*P*] where *P* = (*F*_o_^2^ + 2*F*_c_^2^)/3
*S* = 1.06	(Δ/σ)_max_ = 0.001
4034 reflections	Δρ_max_ = 0.69 e Å^−^^3^
238 parameters	Δρ_min_ = −0.39 e Å^−^^3^
1 restraint	Extinction correction: *SHELXL97* (Sheldrick, 2008), Fc^*^=kFc[1+0.001xFc^2^λ^3^/sin(2θ)]^-1/4^
Primary atom site location: structure-invariant direct methods	Extinction coefficient: 0.0206 (15)

### Special details

**Table:**

Geometry. All e.s.d.'s (except the e.s.d. in the dihedral angle between two l.s. planes) are estimated using the full covariance matrix. The cell e.s.d.'s are taken into account individually in the estimation of e.s.d.'s in distances, angles and torsion angles; correlations between e.s.d.'s in cell parameters are only used when they are defined by crystal symmetry. An approximate (isotropic) treatment of cell e.s.d.'s is used for estimating e.s.d.'s involving l.s. planes.
Refinement. Refinement of *F*^2^ against ALL reflections. The weighted *R*-factor *wR* and goodness of fit *S* are based on *F*^2^, conventional *R*-factors *R* are based on *F*, with *F* set to zero for negative *F*^2^. The threshold expression of *F*^2^ > σ(*F*^2^) is used only for calculating *R*-factors(gt) *etc*. and is not relevant to the choice of reflections for refinement. *R*-factors based on *F*^2^ are statistically about twice as large as those based on *F*, and *R*- factors based on ALL data will be even larger.

### Fractional atomic coordinates and isotropic or equivalent isotropic displacement parameters (Å^2^)

**Table:**

	*x*	*y*	*z*	*U*_iso_*/*U*_eq_	
C1	0.76485 (15)	0.66788 (16)	0.17902 (8)	0.0312 (3)	
C10	0.93118 (13)	0.91852 (15)	−0.08185 (7)	0.0252 (3)	
C11	0.79381 (13)	0.94222 (14)	−0.07596 (7)	0.0216 (3)	
C12	0.71089 (12)	0.83505 (13)	−0.05612 (7)	0.0207 (3)	
C13	0.98070 (14)	1.04447 (16)	−0.09800 (8)	0.0296 (3)	
C14	0.5674 (2)	0.8996 (2)	0.29302 (10)	0.0556 (6)	
C15	0.50380 (15)	0.94048 (18)	−0.09413 (10)	0.0373 (4)	
C16	0.36049 (15)	0.90887 (17)	−0.09383 (10)	0.0354 (4)	
C17	0.88027 (17)	1.27272 (16)	−0.11789 (10)	0.0375 (4)	
C18	0.86633 (18)	1.2985 (2)	−0.19860 (12)	0.0523 (5)	
C19	0.8539 (2)	1.2068 (3)	−0.24945 (12)	0.0708 (7)	
C2	0.73094 (17)	0.75204 (19)	0.23458 (9)	0.0389 (4)	
C3	0.60421 (17)	0.80532 (16)	0.23408 (8)	0.0335 (3)	
C4	0.51129 (15)	0.77163 (14)	0.17715 (8)	0.0283 (3)	
C5	0.54257 (13)	0.68865 (14)	0.12097 (8)	0.0244 (3)	
C6	0.67012 (13)	0.63684 (13)	0.12255 (7)	0.0211 (3)	
C7	0.76898 (13)	0.71167 (13)	−0.04258 (7)	0.0208 (3)	
C8	0.90547 (13)	0.68843 (14)	−0.05067 (8)	0.0256 (3)	
C9	0.98638 (14)	0.78904 (15)	−0.06954 (8)	0.0284 (3)	
H1	0.8499	0.6328	0.1797	0.037*	
H13	1.0678	1.0651	−0.1057	0.036*	
H14A	0.5216	0.9759	0.2711	0.083*	
H14B	0.6461	0.9291	0.3214	0.083*	
H14C	0.5109	0.8538	0.3242	0.083*	
H15A	0.5298	0.9353	−0.1436	0.045*	
H15B	0.5208	1.0310	−0.0761	0.045*	
H16A	0.3429	0.8224	−0.1158	0.053*	
H16B	0.3094	0.9761	−0.1211	0.053*	
H16C	0.3368	0.9077	−0.0444	0.053*	
H17A	0.9628	1.3116	−0.0969	0.045*	
H17B	0.8085	1.3171	−0.0958	0.045*	
H18	0.8666	1.3877	−0.2137	0.063*	
H19A	0.8532	1.1163	−0.2367	0.085*	
H19B	0.8458	1.2318	−0.2984	0.085*	
H1N	0.6049	0.6137	−0.0394	0.026*	
H2	0.7940	0.7731	0.2728	0.047*	
H4	0.4259	0.8057	0.1768	0.034*	
H5	0.4795	0.6678	0.0828	0.029*	
H8	0.9400	0.6025	−0.0429	0.031*	
H9	1.0756	0.7732	−0.0742	0.034*	
N1	0.68677 (11)	0.60110 (11)	−0.02548 (6)	0.0216 (2)	
N2	0.76199 (11)	1.07276 (12)	−0.08760 (7)	0.0254 (3)	
N3	0.87862 (12)	1.13021 (12)	−0.10026 (7)	0.0275 (3)	
O1	0.57901 (9)	0.84573 (10)	−0.04824 (5)	0.0253 (2)	
O2	0.84526 (10)	0.49024 (11)	0.06679 (6)	0.0292 (2)	
O3	0.61092 (10)	0.41953 (10)	0.04911 (6)	0.0286 (2)	
S1	0.70831 (3)	0.52451 (3)	0.053468 (17)	0.02072 (11)	

### Atomic displacement parameters (Å^2^)

**Table:**

	*U*^11^	*U*^22^	*U*^33^	*U*^12^	*U*^13^	*U*^23^
C1	0.0241 (7)	0.0381 (8)	0.0310 (7)	0.0053 (6)	−0.0010 (6)	−0.0003 (6)
C10	0.0198 (6)	0.0297 (7)	0.0264 (6)	−0.0003 (5)	0.0042 (5)	0.0030 (5)
C11	0.0198 (6)	0.0234 (6)	0.0218 (6)	0.0011 (5)	0.0027 (5)	0.0019 (5)
C12	0.0169 (6)	0.0233 (6)	0.0221 (6)	0.0018 (5)	0.0038 (5)	0.0004 (5)
C13	0.0210 (7)	0.0338 (8)	0.0343 (7)	−0.0038 (6)	0.0038 (5)	0.0060 (6)
C14	0.0765 (14)	0.0595 (12)	0.0305 (8)	0.0257 (11)	0.0018 (9)	−0.0124 (8)
C15	0.0246 (7)	0.0411 (9)	0.0460 (9)	0.0066 (6)	0.0024 (6)	0.0175 (7)
C16	0.0217 (7)	0.0376 (8)	0.0463 (9)	0.0036 (6)	−0.0010 (6)	0.0049 (7)
C17	0.0338 (8)	0.0267 (8)	0.0519 (10)	−0.0066 (6)	0.0024 (7)	0.0104 (7)
C18	0.0383 (10)	0.0547 (12)	0.0637 (13)	−0.0004 (8)	0.0034 (9)	0.0353 (10)
C19	0.0733 (16)	0.099 (2)	0.0401 (11)	0.0078 (14)	0.0022 (10)	0.0231 (12)
C2	0.0410 (9)	0.0466 (10)	0.0272 (7)	0.0060 (7)	−0.0072 (6)	−0.0061 (7)
C3	0.0465 (9)	0.0310 (8)	0.0236 (7)	0.0083 (7)	0.0064 (6)	0.0012 (6)
C4	0.0294 (7)	0.0253 (7)	0.0312 (7)	0.0078 (6)	0.0079 (6)	0.0042 (6)
C5	0.0220 (6)	0.0225 (6)	0.0284 (7)	0.0020 (5)	0.0016 (5)	0.0012 (5)
C6	0.0222 (6)	0.0191 (6)	0.0225 (6)	0.0017 (5)	0.0044 (5)	0.0023 (5)
C7	0.0204 (6)	0.0214 (6)	0.0210 (6)	0.0013 (5)	0.0044 (5)	0.0006 (5)
C8	0.0223 (7)	0.0264 (7)	0.0287 (7)	0.0071 (5)	0.0064 (5)	0.0026 (5)
C9	0.0189 (6)	0.0338 (8)	0.0333 (7)	0.0045 (5)	0.0071 (5)	0.0046 (6)
N1	0.0201 (5)	0.0204 (5)	0.0242 (5)	0.0014 (4)	0.0022 (4)	0.0021 (4)
N2	0.0225 (6)	0.0233 (6)	0.0306 (6)	−0.0027 (4)	0.0036 (4)	0.0047 (5)
N3	0.0249 (6)	0.0261 (6)	0.0317 (6)	−0.0052 (5)	0.0024 (5)	0.0063 (5)
O1	0.0169 (5)	0.0249 (5)	0.0346 (5)	0.0035 (4)	0.0052 (4)	0.0079 (4)
O2	0.0237 (5)	0.0299 (5)	0.0345 (5)	0.0111 (4)	0.0053 (4)	0.0055 (4)
O3	0.0317 (5)	0.0183 (5)	0.0362 (5)	−0.0026 (4)	0.0045 (4)	0.0027 (4)
S1	0.02049 (18)	0.01647 (17)	0.02557 (18)	0.00367 (11)	0.00417 (12)	0.00238 (11)

### Geometric parameters (Å, °)

**Table:**

C1—H1	0.9300	C19—H19B	0.9300
C1—C2	1.387 (2)	C19—H19A	0.9300
C10—C9	1.415 (2)	C19—C18	1.304 (4)
C10—C13	1.392 (2)	C2—H2	0.9300
C11—C10	1.4271 (18)	C3—C14	1.505 (2)
C11—C12	1.4257 (18)	C3—C2	1.390 (2)
C11—N2	1.3509 (18)	C4—H4	0.9300
C12—O1	1.3626 (15)	C4—C3	1.386 (2)
C13—H13	0.9300	C5—H5	0.9300
C13—N3	1.340 (2)	C5—C4	1.381 (2)
C14—H14C	0.9600	C6—C5	1.3910 (18)
C14—H14B	0.9600	C6—C1	1.385 (2)
C14—H14A	0.9600	C7—N1	1.4323 (17)
C15—H15B	0.9700	C7—C8	1.4247 (18)
C15—H15A	0.9700	C7—C12	1.3749 (18)
C15—C16	1.488 (2)	C8—H8	0.9300
C15—O1	1.4380 (17)	C8—C9	1.359 (2)
C16—H16C	0.9600	C9—H9	0.9300
C16—H16B	0.9600	N1—H1N	0.8562
C16—H16A	0.9600	N2—N3	1.3528 (16)
C17—H17B	0.9700	S1—C6	1.7600 (13)
C17—H17A	0.9700	S1—N1	1.6358 (11)
C17—C18	1.499 (3)	S1—O3	1.4352 (10)
C17—N3	1.4549 (19)	S1—O2	1.4305 (10)
C18—H18	0.9300		
			
C2—C1—H1	120.5	H19A—C19—H19B	120.0
C6—C1—H1	120.5	C18—C19—H19B	120.0
C6—C1—C2	119.01 (14)	C18—C19—H19A	120.0
C9—C10—C11	120.91 (13)	C3—C2—H2	119.5
C13—C10—C11	103.93 (12)	C1—C2—H2	119.5
C13—C10—C9	135.12 (13)	C1—C2—C3	121.00 (14)
C12—C11—C10	119.89 (12)	C2—C3—C14	121.45 (16)
N2—C11—C10	111.60 (12)	C4—C3—C14	119.87 (15)
N2—C11—C12	128.43 (12)	C4—C3—C2	118.67 (14)
C7—C12—C11	117.44 (12)	C3—C4—H4	119.2
O1—C12—C11	125.18 (12)	C5—C4—H4	119.2
O1—C12—C7	117.36 (12)	C5—C4—C3	121.52 (13)
C10—C13—H13	126.6	C6—C5—H5	120.6
N3—C13—H13	126.6	C4—C5—H5	120.6
N3—C13—C10	106.70 (12)	C4—C5—C6	118.77 (13)
H14B—C14—H14C	109.5	C5—C6—S1	118.97 (10)
H14A—C14—H14C	109.5	C1—C6—S1	119.95 (11)
C3—C14—H14C	109.5	C1—C6—C5	121.02 (13)
H14A—C14—H14B	109.5	C8—C7—N1	119.24 (12)
C3—C14—H14B	109.5	C12—C7—N1	118.49 (11)
C3—C14—H14A	109.5	C12—C7—C8	122.08 (12)
H15A—C15—H15B	108.3	C7—C8—H8	119.3
C16—C15—H15B	109.9	C9—C8—H8	119.3
O1—C15—H15B	109.9	C9—C8—C7	121.45 (13)
C16—C15—H15A	109.9	C10—C9—H9	120.9
O1—C15—H15A	109.9	C8—C9—H9	120.9
O1—C15—C16	109.09 (13)	C8—C9—C10	118.16 (12)
H16B—C16—H16C	109.5	S1—N1—H1N	112.5
H16A—C16—H16C	109.5	C7—N1—H1N	112.9
C15—C16—H16C	109.5	C7—N1—S1	121.19 (9)
H16A—C16—H16B	109.5	C11—N2—N3	103.48 (11)
C15—C16—H16B	109.5	N2—N3—C17	118.53 (12)
C15—C16—H16A	109.5	C13—N3—C17	127.12 (13)
H17A—C17—H17B	107.8	C13—N3—N2	114.27 (12)
C18—C17—H17B	109.1	C12—O1—C15	117.63 (11)
N3—C17—H17B	109.1	N1—S1—C6	108.84 (6)
C18—C17—H17A	109.1	O3—S1—C6	107.93 (6)
N3—C17—H17A	109.1	O2—S1—C6	107.50 (6)
N3—C17—C18	112.65 (15)	O3—S1—N1	104.63 (6)
C17—C18—H18	117.2	O2—S1—N1	108.30 (6)
C19—C18—H18	117.2	O2—S1—O3	119.30 (6)
C19—C18—C17	125.68 (19)		

### Hydrogen-bond geometry (Å, °)

**Table:**

*D*—H···*A*	*D*—H	H···*A*	*D*···*A*	*D*—H···*A*
N1—H1N···O3^i^	0.86	2.21	3.0199 (15)	159.
C8—H8···O2^ii^	0.93	2.44	3.1270 (17)	131.

Symmetry codes: (i) −*x*+1, −*y*+1, −*z*; (ii) −*x*+2, −*y*+1, −*z*.
